# 

**Published:** 1892-03

**Authors:** 


### CONTENTS.

ORIGINAL PAPERS: Page

A Recent Outbreak of Rötheln. By WILLIAM JOHNSTONE FYFFE, M.D. 1

On a Rare Form of Spina Bifida (Syringo-Myelocele). By CHARLES A. MORTON, F.R.C.S. Eng. ... ... ... ... ... ... ... ... ... ... 14

The Quantitative Estimation of the Knee-Jerk. By EWEN J. MACLEAN, M.D., C.M. Edin. ... ... ... ... ... ... ... ... ... ... ... 20

PROGRESS OF THE MEDICAL SCIENCES ... ... ... ... ... ... ... ... 26

REVIEWS OF BOOKS ... ... ... ... ... ... ... ... ... ... ... ... ... 45

NOTES ON PREPARATIONS FOR THE SICK ... ... ... ... ... ... ... ... 57

MEETINGS ... ... ... ... ... ... ... ... ... ... ... ... ... ... ... 59

THE LIBRARY OF THE BRISTOL MEDICO-CHIRURGICAL SOCIETY ... ... ... 62

OBITUARY ... ... ... ... ... ... ... ... ... ... ... ... ... ... ... 67

INTERNATIONAL CONGRESS OF GYNÆCOLOGY AND OBSTETRICS ... ... ... 68

ALVARENGA PRIZE OF THE COLLEGE OF PHYSICIANS OF PHILADELPHIA ... 69

SCRAPS ... ... ... ... ... ... ... ... ... ... ... ... ... ... ... ... 69

EDITORIAL NOTE ... ... ... ... ... ... ... ... ... ... ... ... ... 72

